# Ordered Mesoporous Cu–Co Supported on Al_2_O_3_ Catalysts for Higher Alcohol Synthesis from Syngas: Effect of Cu/Co Ratio on Structure and Performance

**DOI:** 10.3390/nano16080450

**Published:** 2026-04-09

**Authors:** Guoqiang Zhang, Ruiqin Liu, Yuan Zhou, Huayan Zheng, Fanhui Meng

**Affiliations:** 1 School of Resources and Environmental Engineering, Moutai Institute, Zunyi 564507, China; 2 State Key Laboratory of Clean and Efficient Coal Utilization, Taiyuan University of Technology, Taiyuan 030024, China; 3 Experimental Support Center, Moutai Institute, Zunyi 564507, China

**Keywords:** CuCoAl catalysts, ordered mesoporous materials, evaporation-induced self-assembly, higher alcohol synthesis, synergistic effect

## Abstract

CuCo-based catalysts are promising candidates for higher alcohol synthesis from syngas, yet their performance is often limited by poor metal dispersion and insufficient Cu-Co synergy. In this work, a series of ordered mesoporous CuCoAl catalysts with varying Cu/Co atomic ratios were synthesized via the evaporation-induced self-assembly (EISA) method. The structural, electronic, and catalytic properties were systematically investigated using N_2_ physisorption, XRD, TEM, H_2_-TPR, CO-TPD, XPS, and fixed-bed reactor evaluation. The results show that all CuCoAl catalysts prepared by the EISA method possess well-ordered mesoporous structures with high surface areas (up to 235 m^2^/g) and narrow pore size distributions. The interaction between Cu and Co stabilizes the mesoporous framework, inhibits Cu particle growth, and induces electron transfer from Cu to Co as evidenced by XPS. Among the catalysts tested, Cu_1_Co_1_Al (Cu/Co = 1:1) exhibits the highest strong CO adsorption capacity (1.54 mmol/g) and surface hydroxyl content (63.29%), achieving a CO conversion of 32.9% with a C_2_^+^ alcohol space–time yield of 20.5 mg·gcat^−^^1^·h^−1^. These findings establish clear structure–performance relationships for ordered mesoporous CuCoAl catalysts and provide fundamental guidance for the rational design of efficient catalysts for higher alcohol synthesis.

## 1. Introduction

Higher alcohols are widely recognized as promising clean liquid fuels and important intermediates in the chemical industry [1,2]. Traditionally, these alcohols are produced via the hydration of petroleum-derived olefins [3]. However, the depletion of crude oil reserves and growing environmental concerns have driven the search for alternative non-petroleum routes. In this context, direct conversion of syngas (a mixture of H_2_ and CO) derived from coal, natural gas, or biomass into higher alcohols has emerged as a particularly attractive pathway due to its feedstock flexibility and high carbon efficiency [4,5].

The catalytic conversion of syngas to higher alcohols involves a complex reaction network. This network includes multiple elementary steps: adsorption of CO and H_2_ on catalyst surfaces, dissociation of C–O and H–H bonds, formation of C–C and O–H bonds, and stabilization of reaction intermediates [6,7,8]. Such complexity imposes stringent requirements on catalyst design. An ideal catalyst must simultaneously enable two key capabilities: dissociative adsorption of CO followed by hydrogenation to generate CH*_x_* species, and insertion of molecularly adsorbed CO into C–C bonds for chain growth [9,10]. Maintaining an appropriate balance between these functions is critical. Among various catalytic systems, CuCo-based bimetallic catalysts have attracted particular attention. These catalysts operate through a unique dual-site synergistic mechanism. Cu serves as the active site for non-dissociative CO adsorption and H_2_ activation, while Co promotes CO dissociation and C–C coupling [11,12,13]. This synergistic combination positions CuCo catalysts as promising candidates for higher alcohol synthesis.

The dual-site mechanism of CuCo catalysts has been extensively investigated [13,14,15]. Sun et al. [15] and Yang et al. [16] employed infrared spectroscopy to study this mechanism. They demonstrated that higher alcohol synthesis proceeds via a cooperative process. First, dissociatively adsorbed CO on Co sites undergoes hydrogenation to form alkyl CH*_x_* species. These CH*_x_* species subsequently insert into non-dissociatively adsorbed CO on Cu sites, generating acyl intermediates. Further hydrogenation of these intermediates ultimately yields higher alcohol. Prieto et al. [17] reported that the highest ethanol selectivity was achieved at a Cu/Co atomic ratio of 1:2, corresponding to the maximum formation of CuCo alloys. Gao et al. [18] provided further insights into the electronic effects of alloying. Their work revealed that electron transfer occurs from Cu to Co upon alloy formation, decreasing the electron density of Cu species while increasing that of Co species. This electronic modulation enhances CO adsorption on Cu and facilitates weak CO dissociative adsorption on Co, synergistically promoting higher alcohol synthesis.

The choice of support material significantly influences the dispersion of active components and catalytic performance of CuCo catalysts. Various supports, including SiO_2_, Al_2_O_3_, La_2_O_3_, and ZrO_2_, have been investigated for their ability to disperse CuCo species and enhance total alcohol and higher alcohol selectivity [15,19,20,21]. Sun et al. [21] synthesized CuCoM (M = Fe, Cr, Ga, Al) nanoplate catalysts derived from layered double hydroxide precursors via coprecipitation, demonstrating that optimized CuCoAl nanoplates with appropriate surface Cu/Co ratios and enhanced Cu-Co synergistic interactions exhibited significantly improved catalytic performance. Wang et al. [22] compared the effects of Al_2_O_3_, La_2_O_3_, and ZrO_2_ supports on coprecipitated CuCo catalysts, finding that Al_2_O_3_- and ZrO_2_-supported catalysts displayed superior reaction stability. These findings underscore the importance of rational catalyst design in achieving desirable activity and selectivity for CO hydrogenation.

Recent advances in materials synthesis have demonstrated that confining metal nanoparticles within ordered mesoporous structures represents an effective strategy for enhancing catalyst performance [23,24]. The spatial confinement effect of ordered mesopores offers several advantages. It inhibits metal nanoparticle sintering and carbon deposition during reactions. The high surface area facilitates uniform dispersion of active metals. Additionally, the well-defined mesoporous channels provide efficient pathways for reactant transport [25]. Among various mesoporous materials, ordered mesoporous alumina (OMA) has garnered considerable attention as a catalyst support due to its uniform pore architecture, high surface area, and narrow pore size distribution [26,27].

Based on these considerations, this study leverages the structural advantages of ordered mesoporous alumina to address key challenges in CuCo catalyst design. A series of ordered mesoporous Cu-Co/Al_2_O_3_ catalysts with varying Cu/Co atomic ratios were synthesized using the evaporation-induced self-assembly (EISA) method. The catalytic performance of these materials was systematically evaluated for CO hydrogenation to higher alcohols. Through comprehensive characterization, we investigated how the Cu/Co atomic ratio influences catalyst structure, reduction behavior, CO adsorption properties, and Cu-Co interactions. The primary objective is to establish structure–performance relationships. These relationships will provide fundamental insights and practical guidance for the rational design of efficient CuCo-based catalysts for higher alcohol synthesis.

## 2. Materials and Methods

### 2.1. Catalyst Preparation

A series of ordered mesoporous CuCoAl catalysts with different Cu/Co atomic ratios were synthesized using the evaporation-induced self-assembly (EISA) method. In a typical procedure, 1.0 g of triblock copolymer Pluronic F127 (Sigma-Aldrich, St. Louis, MO, USA) was dissolved in 10 mL of anhydrous ethanol (Tianjin Guangfu Fine Chemical Research Institute, Tianjin, China) under ultrasonication to obtain a homogeneous solution (denoted as solution A). Separately, an ethanol solution containing Cu(NO_3_)_2_, Co(NO_3_)_2_, and Al(NO_3_)_3_ (Sinopharm Chemical Reagent Co., Ltd., Shanghai, China) was prepared with a total cation concentration of 1.0 mol/L (denoted as solution B). Solution B was then slowly added to solution A under vigorous stirring. The resulting mixture was continuously stirred at 40 °C for 4 h in a water bath to form a sol. The obtained sol was transferred to a Petri dish and aged at 60 °C for 4 days to allow slow solvent evaporation, yielding a transparent xerogel. The xerogel was subsequently ground into a fine powder and calcined at 400 °C for 5 h in a muffle furnace (ramp rate: 1 °C/min) to remove the template and form the metal oxide framework. The resulting catalysts are denoted as Cu_x_Co_y_Al, where the (Cu + Co)/Al atomic ratio was fixed at 1/9, and the Cu/Co molar ratios (x:y) were varied as 0:1, 1:0, 1:1, 3:1, and 1:2. For comparison, a reference catalyst with the same Cu_1_Co_1_Al composition was prepared by a conventional coprecipitation method following the procedure reported by Wang et al. [22], denoted as Cu_1_Co_1_Al-CP.

### 2.2. Catalyst Characterization

Small-angle X-ray diffraction (XRD) patterns were recorded on a Bruker D8 Advance diffractometer (Karlsruhe, Germany) over a 2θ range of 0.5° to 10° with a scanning rate of 1°/min.

In situ XRD measurements were performed on a Rigaku SmartLab SE diffractometer (Tokyo, Japan) equipped with Cu Kα radiation (λ = 0.1541862 nm), operating at 40 kV and 40 mA. The catalysts were heated from 50 °C to 500 °C under a reducing atmosphere (10% H_2_/N_2_). Spectra were collected over a 2θ range of 5° to 80° at a scanning rate of 10°/min.

Transmission electron microscopy (TEM) images were obtained on a JEM-2100F microscope (JEOL, Tokyo, Japan) operating at an accelerating voltage of 200 kV with a point resolution of 0.19 nm. The morphology and microstructure of the samples were examined under these conditions.

N_2_ physisorption measurements were conducted on a 3H-2000PS2 analyzer (Beishide, Beijing, China) to determine specific surface area, pore volume, and pore size distribution. Prior to analysis, approximately 100 mg of catalyst was degassed under vacuum at 250 °C for 4 h. N_2_ adsorption–desorption isotherms were then recorded at liquid nitrogen temperature. Specific surface areas were calculated using the Brunauer–Emmett–Teller (BET) method, while pore size distributions and pore volumes were determined by the Barrett–Joyner–Halenda (BJH) method.

Hydrogen temperature-programmed reduction (H_2_-TPR) experiments were carried out on an AutoChem 2920 chemisorption analyzer (Micromeritics, Norcross, GA, USA). Approximately 20 mg of catalyst was placed in a U-shaped quartz reactor and pretreated under N_2_ flow (40 mL/min) at 300 °C for 30 min to remove adsorbed impurities and moisture. The sample was then cooled to 50 °C under N_2_ atmosphere. The gas was switched to a 10% H_2_/Ar mixture (30 mL/min), and the temperature was ramped to 900 °C at a heating rate of 10 °C/min after baseline stabilization. Hydrogen consumption was continuously monitored by a thermal conductivity detector (TCD).

Carbon monoxide temperature-programmed desorption (CO-TPD) measurements were performed on the same AutoChem 2920 analyzer ((Micromeritics, Norcross, GA, USA). Approximately 0.1 g of catalyst was loaded into a U-shaped quartz reactor and reduced in a 10% H_2_/Ar flow (30 mL/min) by heating to 300 °C at 10 °C/min and holding for 1 h. The sample was then purged with N_2_ for 0.5 h and cooled to 50 °C. CO adsorption was carried out by introducing a 10% CO/He mixture, followed by Ar purging to remove weakly adsorbed species. After stabilizing the TCD signal, temperature-programmed desorption was performed by heating to 500 °C at 10 °C/min, and the CO desorption signal was recorded by TCD.

X-ray photoelectron spectroscopy (XPS) analysis was conducted on an AXIS ULTRA DLD spectrometer (Kratos, Manchester, UK) equipped with monochromatic Al Kα radiation (hv = 1486.6 eV). The X-ray source was operated at 250 W with an accelerating voltage of 12.5 kV. The analysis chamber pressure was maintained at approximately 2 × 10^−8^ Pa. Binding energies were calibrated using the C 1s peak (284.6 eV) as a reference.

### 2.3. Catalyst Performance Evaluation

Catalytic performance tests were conducted in a fixed-bed reactor system. A total of 1.0 g of catalyst (20–40 mesh) was mixed with 1.0 mL of quartz sand and loaded into a stainless-steel reactor tube with an inner diameter of 8 mm. The catalyst was reduced in situ under atmospheric pressure by flowing a 10% H_2_/N_2_ mixture (50 mL/min) while heating to 300 °C at a ramping rate of 2 °C/min. The reduction was maintained at 300 °C for 4 h. After reduction, the gas was switched to syngas (H_2_/CO = 2:1) with a flow rate of 50 mL/min. The reaction was carried out at 300 °C under a pressure of 6.0 MPa. Catalytic activity data were averaged from measurements taken between 12 and 24 h on stream to ensure steady-state conditions. The reaction products were analyzed using four gas chromatographs. H_2_, CO, CO_2_, and CH_4_ in the gas phase were analyzed online by a GC4000A chromatograph (East & West Analytical Instruments, Beijing, China) equipped with a carbon molecular sieve column and a thermal conductivity detector (TCD). Hydrocarbons and methanol in the gas phase were analyzed by another GC4000A chromatograph equipped with a GDX-403 column and a flame ionization detector (FID). Methanol and water were analyzed by a third GC4000A chromatograph equipped with a GDX-401 column and a TCD. Liquid-phase alcohols were analyzed by a GC-7AG chromatograph equipped with a Chromsorb 101 column and an FID. Data analysis was performed using the normalization method. Liquid products were calibrated using methanol, while gaseous products were calibrated using methane as the reference [28].

## 3. Results

### 3.1. Textural Properties

**Figure 1:**
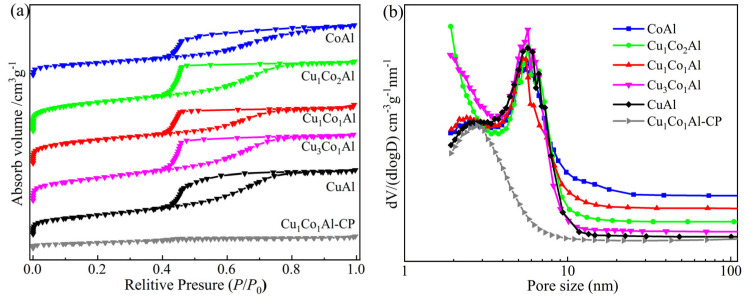
N_2_ adsorption–desorption isotherms (**a**) and pore size distributions (**b**) of the catalysts.

N_2_ physisorption measurements were conducted to examine the textural properties of the catalysts. As shown in Figure 1, all samples prepared by the EISA method exhibited type IV isotherms with H2 hysteresis loops, characteristic of ink-bottle shaped mesopores. The pore size distributions were narrow, with diameters concentrated between 4.1 and 6.1 nm. In contrast, the Cu_1_Co_1_Al-CP catalyst prepared by coprecipitation showed almost no hysteresis loop, suggesting a very low mesopore content.

Table 1 summarizes the textural parameters. The specific surface area followed a bell-shaped trend with increasing Cu content. Notably, all CuCoAl catalysts prepared by the EISA method showed higher surface areas than CuAl and CoAl catalysts. The Cu_1_Co_2_Al catalyst exhibited the largest surface area of 235 m^2^/g, which is favorable for exposing more active sites and facilitating mass transfer during reaction. In contrast, the Cu_1_Co_1_Al-CP catalyst prepared by coprecipitation showed a much lower surface area of only 62 m^2^/g and an average pore diameter of 2.9 nm, indicating the absence of an ordered mesoporous structure.

### 3.2. Structural Analysis

**Figure 2:**
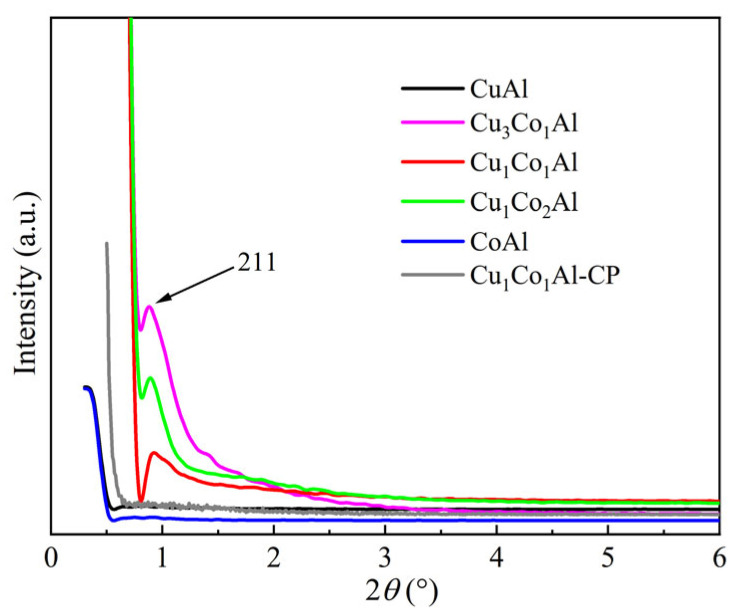
Small-angle XRD patterns of the catalysts.

Small-angle XRD was employed to examine the mesostructural ordering of the catalysts (Figure 2). All CuCoAl catalysts prepared by the EISA method displayed distinct diffraction peaks at approximately 2*θ* = 0.9°, indicating well-ordered mesoporous structures [25]. Among them, Cu_3_Co_1_Al exhibited the most pronounced peak. In contrast, no such diffraction peaks were observed for CuAl and CoAl catalysts, suggesting that the interaction between Cu and Co facilitates the formation of ordered mesopores in ternary metal oxides. Notably, the Cu_1_Co_1_Al-CP catalyst prepared by coprecipitation exhibited no characteristic small-angle diffraction peaks, verifying the absence of ordered mesostructures. This arises from the lack of a structure-directing agent, leading to severe particle aggregation and collapsed, disordered pore channels.

**Figure 3:**
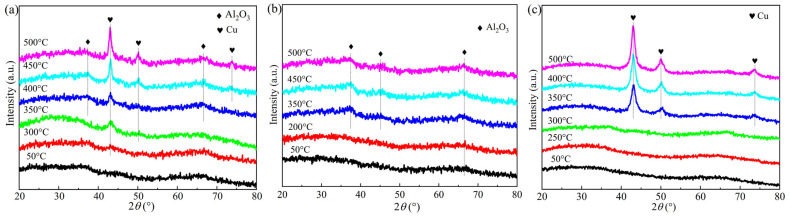
In situ XRD patterns of the catalysts in H_2_ atmosphere at various temperatures (**a**) CuAl; (**b**) CoAl; (**c**) Cu_1_Co_1_Al.

In situ H_2_-XRD was conducted to monitor the structural evolution of CuAl, Cu_1_Co_1_Al, and CoAl catalysts during thermal treatment (Figure 3). For CuAl (Figure 3a), weak diffraction peaks corresponding to metallic Cu (JCPDS No.04-0836) appeared at 300 °C, while for Cu_1_Co_1_Al (Figure 3c), these peaks emerged at 350 °C. The peak intensities increased with temperature for both catalysts. Moreover, no diffraction peaks attributable to Co species were detected in either CoAl or Cu_1_Co_1_Al. The absence of detectable Co diffraction peaks suggests that Co species are either highly dispersed or present as amorphous phases, which is common for Co supported on alumina at these loadings.

The average Cu crystallite sizes were calculated using the Scherrer equation (Table 1). The Cu_1_Co_1_Al catalyst exhibited a smaller Cu crystallite size (10.5 nm) compared with the CuAl catalyst (12.9 nm). Combined with the significantly higher specific surface area of all CuCoAl catalysts prepared by the EISA method and the stable ordered mesoporous structure confirmed by small-angle XRD, this result suggests that the introduction of Co can suppress Cu particle growth and improve the dispersion of active metal species. Smaller Cu particles are generally favorable for catalytic activity in Cu-based hydrogenation reactions [29].

### 3.3. Morphological Analysis

**Figure 4:**
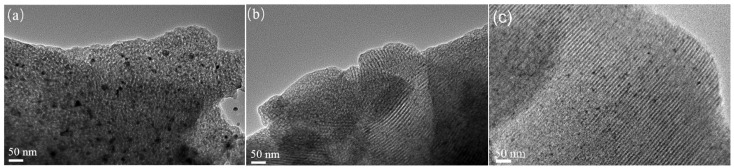
TEM images of the catalysts (**a**) CuAl; (**b**) CoAl; (**c**) Cu_1_Co_1_Al.

Figure 4 shows TEM images of CoAl (a), CuAl (b), and Cu_1_Co_1_Al (c). All catalysts exhibit well-defined ordered mesoporous structures derived from the F127-templated EISA method. In Figure 4a (CuAl), ordered mesopore channels are clearly observed, while Cu particles show relatively large size and slight agglomeration. In Figure 4b (CoAl), the mesoporous framework remains ordered but viewed from a different orientation; no obvious Co-containing particles are observed, indicating that Co species are highly dispersed within the alumina matrix. In Figure 4c (Cu_1_Co_1_Al), highly ordered mesopore channels and uniformly dispersed small metal nanoparticles are clearly visible. The particle size in Cu_1_Co_1_Al is significantly smaller than that in CuAl, confirming that the Cu-Co interaction and mesoporous confinement effect effectively suppress particle growth and enhance metal dispersion. These observations demonstrate that the synergistic interaction between Cu and Co promotes the formation of stable ordered mesopores and improves the dispersion of active metals.

### 3.4. Reduction Behavior

**Figure 5:**
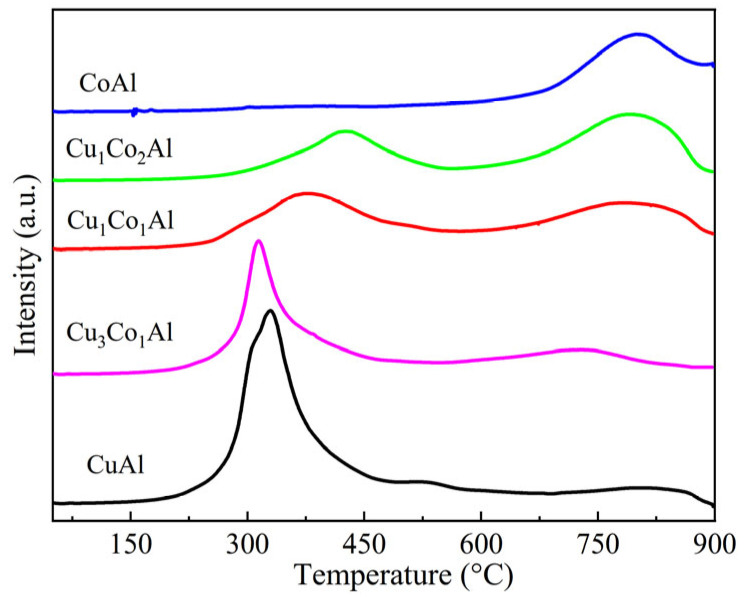
H_2_-TPR profiles of the catalysts.

To further investigate the interaction between Cu and Co, H_2_-TPR measurements were performed on CuAl, CoAl, and CuCoAl catalysts (Figure 5). The CuAl catalyst exhibited a low-temperature reduction peak at 327 °C, attributed to the reduction of CuO [30]. For CoAl, a high-temperature reduction peak was observed at 798 °C, corresponding to the reduction of Co species with high oxidation states [31]. For CuCoAl catalysts, the reduction temperature of Cu species gradually increased with increasing Co content. Meanwhile, the reduction peaks of Co species also shifted toward higher temperatures. This behavior is attributed to the mutual dispersion effect between Cu and Co. During the one-pot EISA synthesis process, Cu and Co precursors are homogeneously mixed at the molecular level and incorporated into the mesoporous alumina framework, where they act as spatial barriers for each other to inhibit agglomeration during calcination and reduction. This enhanced mutual dispersion strengthens both the metal–support interaction and the intimate contact between Cu and Co species, thus increasing their reduction temperatures. Similar phenomena have been widely reported in CuCo-based catalytic systems for higher alcohol synthesis [14,32]. These results confirm the strong synergistic interaction between Cu and Co in our catalysts.

### 3.5. CO-TPD Analysis

**Figure 6:**
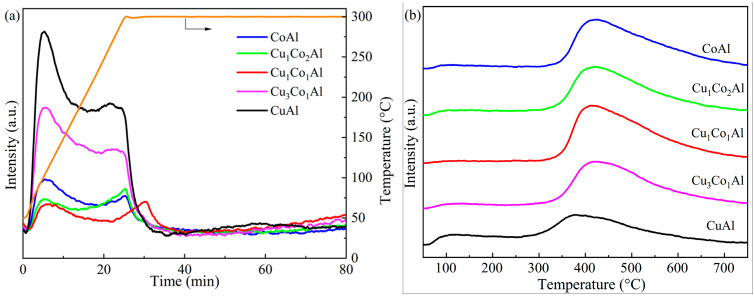
CO desorption profiles of the catalysts: (**a**) Low-temperature range (50–300 °C); (**b**) Full temperature range (50–700 °C).

Figure 6 shows the CO adsorption properties of the catalysts, and the corresponding CO desorption amounts are summarized in Table 2. Figure 6a presents the CO desorption profiles of all catalysts in the low-temperature range of 50–300 °C, which clearly shows the weak adsorption desorption peaks of each sample; Figure 6b shows the full-range CO desorption profiles in the temperature range of 50–700 °C, focusing on the medium and strong CO adsorption behavior of the catalysts. The desorption peaks in the low-temperature region (50–200 °C, centered at 96–99 °C) correspond to physically adsorbed or weakly chemically adsorbed CO species on the catalyst surface, while the desorption peaks in the high-temperature region (300–600 °C) are attributed to strongly chemically adsorbed CO. It is widely accepted that the synthesis of C_2_^+^ alcohol is primarily determined by moderate or strong CO adsorption rather than weak adsorption, as weakly bound CO is prone to direct desorption and cannot participate in the C–C chain growth and CO insertion reactions required for higher alcohol formation [33].

As shown in Table 2, the low-temperature CO desorption amount gradually decreased with decreasing Cu content. CuAl exhibited the highest low-temperature CO desorption (0.162 mmol/g), while CoAl showed the lowest (0.022 mmol/g). In the high-temperature region, significant differences were observed among the catalysts. CuAl displayed the lowest high-temperature CO desorption (0.71 mmol/g) at 380 °C. CoAl showed a much larger desorption amount (1.46 mmol/g) at a higher temperature (426 °C), indicating stronger CO binding on Co sites [23]. For CuCoAl catalysts prepared by the EISA method, the high-temperature CO desorption first increased and then decreased with increasing Co content. Cu_1_Co_1_Al exhibited the maximum CO desorption of 1.54 mmol/g at 424 °C, surpassing both CuAl and CoAl. These results demonstrate that the interaction between Cu and Co significantly enhances strong CO adsorption. The optimal Cu/Co ratio (1:1) maximizes this effect, promoting the formation of surface C* species essential for C–C chain growth and higher alcohol synthesis [13].

### 3.6. XPS Analysis

**Figure 7:**
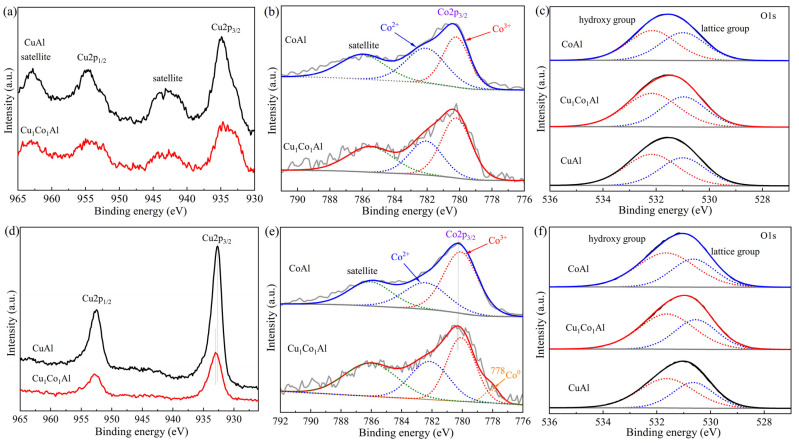
XPS spectra of calcined catalysts (**a**) Cu 2p, (**b**) Co 2p_3/2_, (**c**) O 1s and reduced catalysts (**d**) Cu 2p, (**e**) Co 2p_3/2_, (**f**) O 1s. In subfigures (**b**,**c**,**e**,**f**), the solid lines are the original experimental spectra, and the dashed lines are the fitted component peaks.

XPS was employed to investigate the chemical states and electronic interactions between metals in the catalysts. Figure 7a shows the Cu 2p spectra of calcined catalysts. All samples exhibited two main peaks at approximately 954 eV and 934 eV, corresponding to Cu 2p_1/2_ and Cu 2p_3/2_, respectively, along with prominent satellite peaks at higher binding energies. These features indicate that Cu species primarily existed as CuO in the calcined state [34]. After reduction (Figure 7d), the Cu 2p_3/2_ peaks shifted to lower binding energies (~932 eV) and the satellite peaks disappeared, confirming the reduction of Cu^2+^ to metallic Cu^0^ [14]. Notably, the Cu 2p_3/2_ binding energy of the reduced Cu_1_Co_1_Al catalyst was 0.23 eV higher than that of the reduced CuAl catalyst. This upshift in binding energy confirms the electron transfer from Cu to Co, which is consistent with the formation of Cu-Co interfacial sites.

Figure 7b,e display the Co 2p_3/2_ spectra of calcined and reduced catalysts. The main peak at ~780.2 eV with a satellite feature at ~786.0 eV was observed for all samples. Peak fitting revealed two components, Co^3+^ at ~780.1 eV and Co^2+^ at ~782.2 eV [14], with no significant binding energy differences between catalysts. After reduction, the Cu_1_Co_1_Al catalyst exhibited a distinct Co^0^ peak at 778.0 eV [14], accounting for 4.5% of the total surface Co species. In contrast, no Co^0^ was detected in the reduced CoAl catalyst, indicating that the presence of Cu facilitates the reduction of Co species. This small amount of surface Co^0^ can form abundant Cu-Co interfacial active sites, which is the core of the dual-site synergistic effect for higher alcohol synthesis [17,18]; the remaining Co species in oxidized state also act as structural promoters to enhance the dispersion of Cu species and stabilize the mesoporous framework. Additionally, the Co 2p_3/2_ binding energy of reduced Cu_1_Co_1_Al was slightly higher (0.07 eV) than that of reduced CoAl, suggesting increased surface electron density and enhanced Cu-Co interaction.

The O 1s spectra of calcined and reduced catalysts are shown in Figure 7c,f. Two types of surface oxygen species were identified via peak fitting: hydroxyl groups (OH) at 531.6–532.0 eV and lattice oxygen (O^2−^) at 530.4–530.8 eV [15]. No significant binding energy shifts were observed between catalysts. Quantitative analysis of surface oxygen species is summarized in Table 3. After reduction, all catalysts showed increased hydroxyl group content and decreased lattice oxygen content, indicating that lattice oxygen reacted with hydrogen to form hydroxyl groups during reduction. Notably, Cu_1_Co_1_Al exhibited the highest hydroxyl content among calcined catalysts and showed the greatest conversion of lattice oxygen to hydroxyl groups after reduction. Abundant surface hydroxyl groups promote the formation of CH*_x_* intermediates, which are beneficial for C_2_^+^ alcohol synthesis [12]. These results suggest that Cu-Co interaction enhances lattice oxygen activation, leading to increased hydroxyl group formation.

### 3.7. Catalytic Performance

The catalytic performance of different catalysts for CO hydrogenation to higher alcohols was evaluated in a fixed-bed reactor, and the results are summarized in Table 4. The CuAl and CoAl catalysts exhibited relatively low CO conversions of 12.1% and 3.4%, respectively. CoAl predominantly produced hydrocarbons, with the lowest total alcohol selectivity of 13.0%. In contrast, CuAl favored alcohol formation, achieving a total alcohol selectivity of 27.6%, although methanol accounted for 87.4% of the alcohol distribution, resulting in a low C_2_^+^ alcohol space–time yield (STY) of 2.3 mg·gcat^−1^·h^−1^.

All CuCoAl catalysts prepared by the EISA method showed enhanced catalytic activity compared to their monometallic counterparts. As the Cu content increased, CO conversion first increased and then decreased. Total alcohol selectivity gradually increased with increasing Cu content, while ethanol selectivity and C_2_^+^ alcohol STY exhibited volcanic trends. Among the catalysts tested, Cu_1_Co_1_Al (Cu/Co = 1:1) displayed the best catalytic performance, achieving a CO conversion of 32.9%, a total alcohol STY of 59.4 mg·gcat^−1^·h^−1^, and a C_2_^+^ alcohol STY of 20.5 mg·gcat^−1^·h^−1^. The alcohol distribution over this catalyst showed a significant shift toward higher alcohols, with ethanol, propanol, and butanol accounting for 16.8%, 10.2%, and 6.0% of the total alcohols, respectively. Notably, the Cu_1_Co_1_Al-CP catalyst synthesized by coprecipitation without ordered mesoporous structure showed a CO conversion of only 18.3%, and a C_2_^+^ alcohol STY of 11.2 mg·gcat^−1^·h^−1^, which were significantly lower than those of the ordered mesoporous Cu_1_Co_1_Al catalyst. This result directly confirms that the ordered mesoporous structure plays a crucial role in boosting catalytic activity and C_2_^+^ alcohol synthesis efficiency.

## 4. Discussion

The characterization results demonstrate that the interaction between Cu and Co plays a decisive role in determining both the structural properties and catalytic performance for higher alcohol synthesis. Small-angle XRD results show that only CuCoAl catalysts with Co addition can form stable well-ordered mesoporous structures, while CuAl and CoAl catalysts exhibit poorly ordered mesostructures, indicating that Co species can stabilize the mesoporous framework during the EISA synthesis and high-temperature calcination process. BET results show that all CuCoAl catalysts have significantly higher specific surface areas (202–235 m^2^/g) than CuAl (170 m^2^/g), demonstrating that Co addition can improve the dispersion of active metal species. In situ XRD further showed that Co incorporation inhibits Cu particle growth (10.5 nm for Cu_1_Co_1_Al vs. 12.9 nm for CuAl), demonstrating that Co suppresses the sintering and growth of Cu particles during high-temperature reduction. Combined with H_2_-TPR results that the reduction temperature of Cu species shifts to higher values with Co addition—which reflects enhanced metal–support interaction between active metals and the alumina matrix, as well as improved thermal stability of the catalyst structure—these results collectively confirm that Co acts as a structural promoter in this catalytic system.

XPS analysis provided direct evidence for electronic interaction between Cu and Co. The reduced Cu_1_Co_1_Al exhibited a 0.23 eV upward shift in Cu 2p_3/2_ binding energy relative to CuAl, while Co^0^ species (accounting for 4.5% of surface Co species) were detected only in the presence of Cu. For XPS characterization, the binding energy of core-level electrons is directly determined by the outer electron density of the target atom. An upward shift in binding energy indicates electron loss and decreased electron density of the atom, while a downward shift corresponds to electron gain and increased electron density. Our XPS results thus directly confirm electron transfer from Cu to Co at the bimetallic interface, which creates modified interfacial sites with optimized electronic properties. The “electronic properties” herein specifically refer to three core intrinsic properties of the active sites regulated by this Cu-Co electron transfer: the electron density of Cu and Co dual active sites, the interfacial synergistic interaction between the two metals, and the metal–support interaction between active species and the mesoporous Al_2_O_3_ framework. These electronic properties collectively determine the CO adsorption and activation capacity of the catalyst, balance the CO dissociation and insertion reactions required for C–C chain growth in higher alcohol synthesis, and are directly correlated with the final catalytic performance [32,35].

CO-TPD revealed that Cu_1_Co_1_Al exhibited the highest strong CO adsorption capacity (1.54 mmol/g), surpassing both CuAl (0.71 mmol/g) and CoAl (1.46 mmol/g). This enhancement is attributed to the unique Cu-Co interfacial sites created by optimal metal ratio. O 1s XPS showed that Cu_1_Co_1_Al possessed the highest surface hydroxyl content (63.29% after reduction), with the greatest conversion of lattice oxygen to hydroxyl groups. Surface hydroxyls are known to promote CH*_x_* intermediate formation, essential for C–C chain growth [12,15,36].

Activity tests showed a clear volcanic trend with Cu/Co ratio. Cu_1_Co_1_Al exhibited the best performance: CO conversion of 32.9%, total alcohol STY of 59.4 mg·gcat^−1^·h^−1^, and C_2_^+^ alcohol STY of 20.5 mg·gcat^−1^·h^−1^. The alcohol distribution shifted significantly toward higher alcohols, with C_2_^+^ alcohols accounting for 34.5% of total alcohols (excluding methanol).

Beyond the Cu-Co ratio modulation, the well-ordered mesoporous structure is another core factor determining the superior catalytic performance, which is directly verified by the comparison between the EISA-synthesized Cu_1_Co_1_Al and the coprecipitated Cu_1_Co_1_Al-CP control catalyst with identical chemical composition. Small-angle XRD and N_2_ physisorption confirmed that Cu_1_Co_1_Al-CP lacks ordered mesostructure, with a specific surface area (62 m^2^/g) less than 1/3 of Cu_1_Co_1_Al (206 m^2^/g). The ordered mesoporous framework enhances active component dispersion via spatial confinement, exposes more Cu-Co interfacial sites, and optimizes reactant/product mass transfer. As a result, Cu_1_Co_1_Al achieved 1.8-fold higher CO conversion and C_2_^+^ alcohol STY than Cu_1_Co_1_Al-CP, directly verifying the critical role of mesoporosity in the catalytic system.

## 5. Conclusions

In summary, this work demonstrates that ordered mesoporous Cu-Co/Al_2_O_3_ catalysts with tunable Cu/Co ratios can be successfully synthesized via the EISA method. The Cu-Co interaction plays a crucial role in stabilizing the mesoporous structure, enhancing metal dispersion, and modifying electronic properties. An optimal Cu/Co ratio of 1:1 maximizes strong CO adsorption capacity, surface hydroxyl group concentration, and Cu-Co synergy, leading to superior catalytic performance for higher alcohol synthesis. These findings provide fundamental insights into the design of efficient catalysts and establish clear structure–performance relationships that can guide future catalyst development for syngas conversion to higher alcohols.

## Figures and Tables

**Table 1:** Textural properties of the catalysts.

Catalysts	*S*_BET_ (m^2^/g) ^a^	*V* (cm^3^/g) ^b^	APD (nm) ^c^	*d*_Cu_ (nm) ^d^
CuAl	170	0.23	5.39	12.9
Cu_3_Co_1_Al	202	0.24	4.68	--
Cu_1_Co_1_Al	206	0.21	4.12	10.5
Cu_1_Co_2_Al	235	0.24	4.21	--
CoAl	118	0.18	6.16	--
Cu_1_Co_1_Al-CP	62	0.05	2.90	--

^a^ Calculated using the BET equation. ^b^ BJH desorption pore volume. ^c^ BJH desorption average pore diameter. ^d^ Cu crystallite size calculated from the Cu (111) diffraction peak in the in situ XRD pattern at 500 °C via Scherrer equation.

**Table 2:** CO desorption amount of the catalysts.

Catalysts	Temperature (°C)	CO Desorption (mmol/g)	Temperature (°C)	CO Desorption (mmol/g)
CoAl	96	0.022	426	1.46
Cu_1_Co_2_Al	99	0.024	415	1.23
Cu_1_Co_1_Al	99	0.054	424	1.54
Cu_3_Co_1_Al	98	0.125	427	1.18
CuAl	96	0.162	380	0.71

**Table 3:** Surface oxygen species concentrations (at%) for calcined and reduced catalysts determined by XPS.

Catalysts	Calcined Catalysts		Reduced Catalysts	
	Hydroxyl Group	Lattice Oxygen	Hydroxyl Group	Lattice Oxygen
CoAl	52.09	47.91	57.14	42.86
Cu_1_Co_1_Al	53.48	46.52	63.29	36.71
CuAl	52.82	48.18	57.47	42.53

**Table 4:** Catalytic performance of different catalysts for CO hydrogenation to higher alcohols.

Catalysts	X_CO_%	Selectivity (mol%)					Alcohols Distribution (mol%)					STY (mg·g_cat_^−1^·h^−1^)	
		CH_4_	CO_2_	C_2+_H	DME	ROH	MeOH	EtOH	PrOH	BuOH	C_5+_OH	ROH	C_2+_OH
CuAl	12.1	28.1	41.3	1.9	1.08	27.6	87.4	8.5	2.5	1.3	0.3	33.4	2.3
Cu_3_Co_1_Al	16.4	35.5	12.9	29.5	1.35	21.7	79.6	13.3	4.2	2.3	0.6	35.6	6.4
Cu_1_Co_1_Al	32.9	45.3	6.5	28.6	2.2	17.4	65.5	16.8	10.2	6.0	1.5	59.4	20.5
Cu_1_Co_2_Al	25.4	47.4	8.8	24.6	2.6	16.6	64.8	13.5	12.5	8.0	1.2	42.2	15.0
CoAl	3.4	52.2	4.0	30.6	0.2	13.0	75.9	10.1	3.9	9.1	1.0	8.1	2.0
Cu_1_Co_1_Al-CP	18.3	50.7	5.5	29.1	1.2	13.5	70.6	14.2	9.3	4.7	1.2	38.5	11.2

Reaction conditions: T = 300 °C, P = 6.0 MPa, GSHV = 3000h^−1^, n(H_2_):n(CO) = 2:1.

## Data Availability

The original contributions presented in this study are included in the article. Further inquiries can be directed to the corresponding authors.
